# Genistein Exposure Interferes with Pharmacokinetics of Celecoxib in SD Male Rats by UPLC-MS/MS

**DOI:** 10.1155/2017/6510232

**Published:** 2017-12-04

**Authors:** Xiang Zheng, Jian Wen, Teng-hui Liu, Qiu-Geng Ou-yang, Jian-ping Cai, Hong-yu Zhou

**Affiliations:** School of Pharmaceutical Science, Wenzhou Medical University, Wenzhou, Zhejiang, China

## Abstract

**Objective:**

To discuss the effects of genistein on the metabolism of celecoxib in vitro and in vivo.

**Method:**

In vitro, the effects of genistein on the metabolism of celecoxib were studied using rat and human liver microsomes. In vivo, pharmacokinetics of celecoxib was evaluated in rats with or without genistein. Fifteen Sprague-Dawley (SD) rats were randomized into three groups: celecoxib (A group), celecoxib and 50 mg/kg genistein (B group), and celecoxib and 100 mg/kg genistein (C group). Single dose of 33.3 mg/kg celecoxib was orally administered 30 min after genistein ig. At 0.5, 1, 2, 3, 4, 6, 8, 10, 12, and 24 h after celecoxib administration, 300–400 µl blood samples were collected and the concentration of celecoxib was analyzed by ultrahigh-performance liquid chromatography-tandem mass spectrometry system.

**Result:**

Genistein showed notable inhibitory effects on three microsomes. It affected pharmacokinetics of celecoxib in vivo experiments. Genistein had dramatically ability to suppress CYP2C9∗1 and ∗3. After pretreatment with genistein, AUC and *C*_max_ of the C group were higher than B group. CL_z_/F of C group was lower than the B group.

**Conclusion:**

Genistein inhibits the conversion of celecoxib in vitro and in vivo. So, the dosage of celecoxib should be adjusted if it was used associated with genistein.

## 1. Introduction

**Figure 1:**
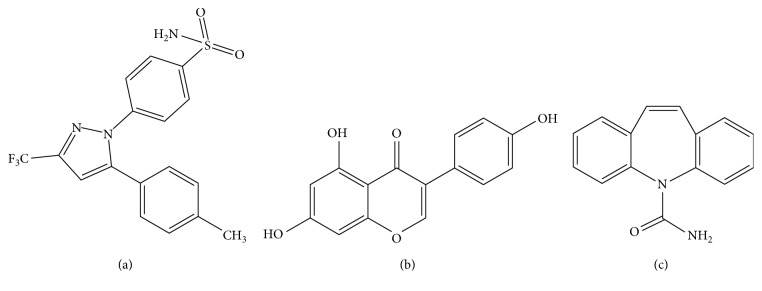
Chemical structures of three substances: (a) genisten, (b) celecoxib, and (c) IS.

Celecoxib (Figure 1), a selective cyclooxygenase-2 (COX-2) inhibitor, has been widely used to treat arthritis for many years [1, 2]. Recently, it was reported to be useful against breast cancers as an adjuvant drug [3–6]. Being a second-generation NSAID, celecoxib is potential to induce adverse effects: hepatic and renal insufficiency [2, 7, 8]. According to DrugBank, the half-time of celecoxib is 11 h in healthy volunteers. Because of its 11 h half-time, the pharmacokinetic progress of celecoxib is more likely to be affected by some food or herbal preparation [9], which might be self-ingested by patients.

Genistein (Figure 1), a kind of isoflavonoids, is mostly derived from soy products and plants. In many reports, soy production shows potential efficacy to reduce the risk of osteoporosis, cardiovascular disease, and cancer [10–12]. Most Asians like to eat soy products as a part of daily necessary [13, 14]. Studies by Burnett et al. and Kopecna-Zapletalova et al. showed that genistein noncompetitively inhibited cytochrome P450 (CYP450) 2C9 and 3A4 [15, 16]. So, we deduce that genistein is a possible origin of many drug interactions by interfering with CYP450.

As is known to all, CYP2C9 and CYP3A4 play an important role in the drug metabolism. CYP2C9 is involved in 15–20% of drug conversion [17]. 3A4 is most plentiful in human liver and participates in 50% drug metabolism [18]. In vivo, celecoxib is oxidized by CYP2C9 and CYP3A4 to the inactive metabolite hydroxycelecoxib, and then hydroxycelecoxib is converted to carboxycelecoxib and celecoxib glucuronide [19]. In this metabolic process, CYP2C9 plays a more important role than 3A4. So, we speculated that mutation in the gene of CYP2C9 may result in altered pharmacokinetics of celecoxib. As we all know, CYP2C9 had gene polymorphism (http://www.cypalleles.ki.se/cyp2c9.htm). CYP2C9∗3 was most frequently present in the white with 15%, and in Han Chinese, this allele variant is present in 3% [20]. The global population base was large even if the proportion of gene mutation was less; this research has a certain degree of meaning.

Currently, the interaction between drug and isoflavones causes a lot of attention. In this study, we want to determine whether genistein influences conversion of celecoxib or not. We hypothesized that genistein might inhibit CYP450 to influence the pharmacokinetics of celecoxib in vivo. Based on this, two experiments were designed: (1) in vitro, the effects of genistein on rat and human liver microsomes (RLM and HLM) and CYP2C9 recombinant enzyme were studied and (2) in vivo, the effects of genistein on pharmacokinetics of celecoxib in male SD rats were detected by ultrahigh-performance liquid chromatography-mass spectrometry (UPLC-MS/MS) method.

## 2. Materials and Methods

### 2.1. Chemicals and Reagents

Celecoxib was purchased from Perfermker (Shanghai, China); genistein and carbamazepine (IS) were purchased from J&K Chemical (Beijing, China); and carboxy methylcellulose sodium salt (CMC), acetonitrile (ACN), formic acid, and other MS-using chemicals were purchased from Sigma-Aldrich Company (Shanghai, China). The primary standard stock solution of celecoxib (1 mg/ml) and IS (500 ng/ml) used in in vitro experiments were prepared by dissolving in methanol. Celecoxib and genistein used in the in vivo experiment were dissolved by 5% CMC.

The male Sprague-Dawley (SD) rats (240 ± 30 g) were purchased from Shanghai Laboratory Animal Center (SLAC). Before starting experiments, they were raised for three weeks in laboratory to minimize the influence of transportation process.

### 2.2. In Vitro Experiments

1.6 µl genistein was added into incubation system which contained 3.81 µl celecoxib (50 µM), 10 µl RLM, HLM, or CYP2C9 allele with b5, 10 µl NADPH, and 174.59 µl 1M PBS. In the first step for determining the inhibitory effect of genistein on celecoxib in HLM and RLM, the experiment was designed to use 100 µM genistein to inhibit celecoxib. The second step was aimed to get IC50, so the concentration of genistein was designed as 0.01, 0.1, 5, 10, 20, 50, and 100 µM. The sample was transported to a −80°C freezer after 1 h incubation.

### 2.3. In Vivo Experiments

15 male SD rats were randomly divided into 3 groups (*n* = 5): celecoxib (A group), celecoxib and single dose of 50 mg/kg genistein (B group), celecoxib and single dose of 100 mg/kg genistein (C group). Single dose of 33.3 mg/kg celecoxib was orally administered 30 min after genistein ig. At 0.5, 1, 2, 3, 4, 6, 8, 10, 12, and 24 h after celecoxib administration, 300–400 µl blood samples were collected into a heparin-treated tube from the tail vein. Samples were immediately centrifuged at 13,000 rpm for 10 min, and then the plasma was transferred to another clean 0.5 ml tube and kept at −80°C in a freezer.

### 2.4. Sample Preparation

100 µl plasma was added into a 1.5 ml tube with 20 µl IS (500 ng/ml) and 300 µl ACN. After vortex mixing for 2 min, samples were centrifuged at 13,000 rpm for 10 min. Supernatant was 1 : 1 diluted into a clean tube with water. 2 µl mixture was injected into UPLC-MS/MS for analysis.

### 2.5. UPLC-MS/MS Method

The concentration of celecoxib was measured by UPLC-MS/MS. The UPLC system comprised a binary solvent manager (BSM) and a sample manager with flow-through needle (SM-FTN) using UPLC®BEH C18 column (2.1 × 50 mm, 1.7 μm; Waters, USA). Mass spectrometer comprised Waters XEVO TQD triple-quadrupole (Waters Corp.) with an electrospray ionization source. Data acquisition and control of the instrument were performed by Masslynx 4.1 software (Waters Corp., Milford, MA, USA). The method selected 0.1% formic acid (a) and ACN (b) as the mobile phase. The proportion of the mobile phase was 40% (a) : 60% (b) during 0–0.5 min. *b* was linearly increased to 95% at 1.5 min, and *b* was decreased to 60% again at 2.0 min. The flow rate was 0.4 ml/min. The total run time was 2.5 min. Celecoxib and IS were analyzed using the MRM method. The mode of MRM was the positive ion mode. In the MRM method, cone voltages were set at 60 V for celecoxib and 40 V for IS. The collision voltage of celecoxib and IS was set at 40 V and 20 V, respectively. Ion mass spectrometric analysis of celecoxib and IS was m/z 381.7–362.2 and 237.1–194.2, respectively.

### 2.6. Statistical Analysis

The pharmaceutics parameters were analyzed by DAS 3.0. The enzyme model was made by Graphpad Prism v5.0. Statistical evaluation of data was counted as mean ± SD using one-way ANOVA by Dunnett's multiple range test (SPSS 18.0). *P* < 0.05 was mean significant.

## 3. Result

### 3.1. UPLC-MS/MS

**Figure 2:**
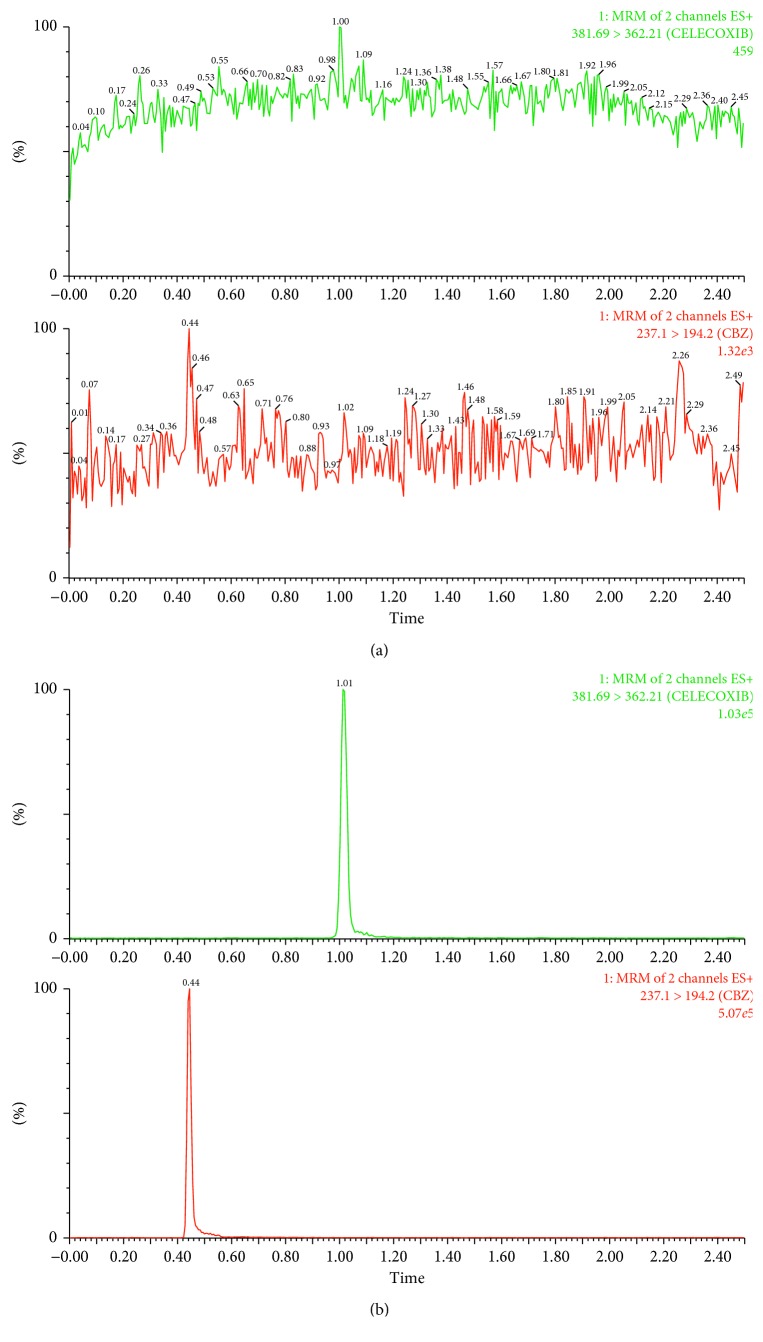
UPLC-MS/MS chromatograph of celecoxib and IS. (a) Blank plasma sample. (b) Genistein-treated rat plasma sample at 10 h after celecoxib.

Chromatogram of celecoxib and IS is shown in Figure 2. Figure 2 shows the blank plasma sample. B showed genistein-treated rat plasma sample at 10 h after celecoxib. Retention time was 1.03 min for celecoxib and 0.45 min for IS. The calibration curve of concentration of celecoxib ranged from 2 to 10,000 ng/ml. From this curve, the calculated correlation coefficient was 0.99.

### 3.2. Effects of Genistein on Celecoxib Conversion Mediated by CYP2C9, RLM, and HLM In Vitro

**Figure 3:**
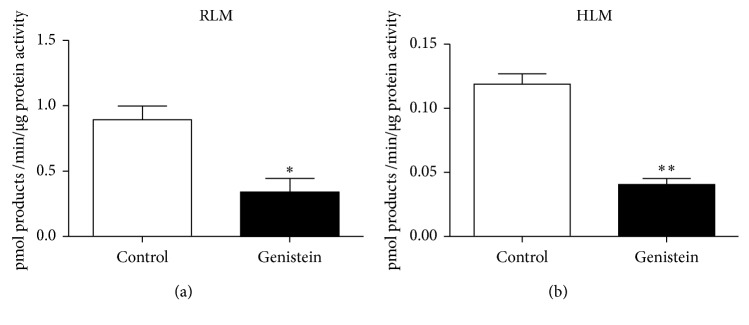
Effects of 100 µM genistein on converting celecoxib.

**Figure 4:**
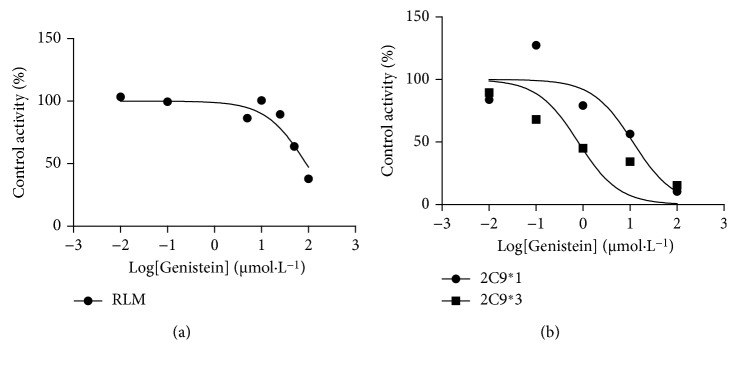
IC50 of genistein on celecoxib in 2C9∗1, 2C9∗3, and RLM.

Figure 3 shows the effects of 100 µM genistein on converting celecoxib by RLM (3.44 mg/ml) and HLM (12.65 mg/ml). Genistein showed notable inhibitory effects on human (34%) and rat liver microsomes (38%) in vitro.  Figure 4 shows IC50 of genistein on celecoxib in 2C9∗1, 2C9∗3, and RLM. Human recombinant CYP2C9 was gained as a gift from Beijing Hospital and Beijing Institute of Geriatrics. The dose-dependent inhibition of genistein on RLM, CYP2C9∗1, and CYP2C9∗3 was 89.94 µM, 11.52 µM, and 0.78 µM, respectively.

### 3.3. Effects of Genistein on Celecoxib Pharmacokinetics in Male SD Rats

**Figure 5:**
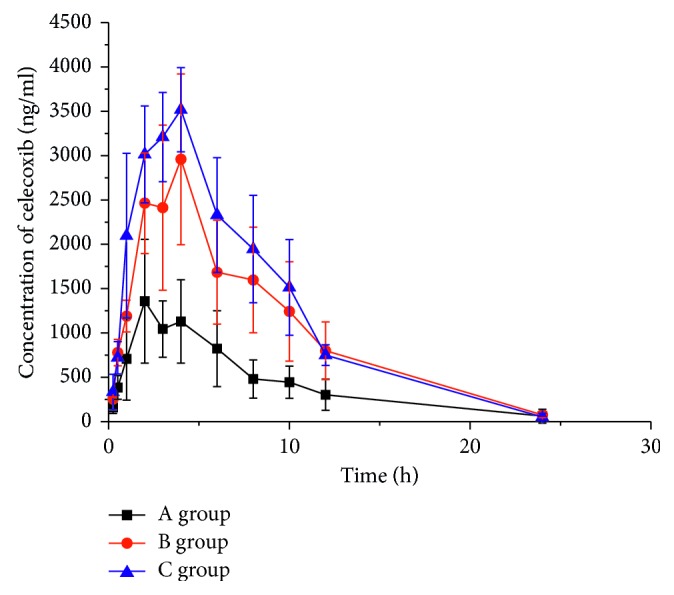
The mean plasma concentration-time curves of celecoxib.

The mean plasma concentration-time curves of celecoxib are presented in Figure 5, and their main pharmacokinetic parameters are presented in Table 1. Pretreated by single dose of genistein, CL_z_/F significantly decreased in the B and C groups (2.03 ± 0.52 and 1.64 ± 0.20 versus 3.49 ± 1.37 l/kg·h; B and C groups versus A group). Correspondingly, AUC_(0–*t*)_, AUC_(0–*∞*)_, and *C*_max_ in the B and C groups increased dose-dependently (AUC_(0–*t*)_: 25.68 ± 7.19 and 30.58 ± 3.59 versus 10.8 ± 5.56 mg/l·h; AUC_(0–*∞*)_: 26.10 ± 7.19 and 30.84 ± 3.71 versus 10.8 ± 5.56 mg/l·h; *C*_max_: 3.24 ± 0.61 and 3.76 ± 0.26 versus 1.38 ± 0.77 mg/l; B and C groups versus A group).

## 4. Discussion

According to the literature, celecoxib might cause hepatotoxicity by influencing CYP2C9 [7, 21]. Celecoxib had three metabolites. CYP2C9 and CYP3A4 were responsible for first steps. In Paulson et al.'s research, they showed that the metabolism of celecoxib in humans is similar to that in rats occurring primarily through a single metabolic pathway with the formation of the methylhydroxy (M3) and carboxylic (M2) acid metabolites [22, 23]. The pharmacokinetic of celecoxib could be influenced by drugs or food. Toxicity of celecoxib was increased when the CYP2C9 inhibitor gefitinib was combined [24]. The possibility of vomiting the celecoxib adjunct with medicine was increased (paracetamol and endone) [25]. These literatures indicated that the adverse effect of celecoxib increased by drug interaction.

The flavone structure of genistein (4′,5,7-trihydroxyisoflavone) suppressed CYP2C9 by connecting with the active site of 2C9 [26]. Genistein had inhibition in vitro and in vivo was verified by many literatures. Given 25 µM genistein, CYP2C9 activity was relatively small (13%) [15]. Genistein could decrease the ability of flurbiprofen being converted to 4′-hydroxylation in vitro [26]. Metabolism of cholecalciferol was decreased by genistein in women [15]. In further research, it was found that genistein had noncompetitive inhibition on CYP2C9 and CYP3A4 [16]. Knowledge on the contribution of genistein with celecoxib in metabolic progress is urgent, and it may lead to dose adjustment aimed at effective therapeutic depth.

Asians have a habit of taking soy products as a daily necessary. Messina et al. summed that the daily per capita intake of soy protein of China, Japan, and Democratic People's Republic of Korea was 2.7 g, 8.7 g, and 9.6 g, which was equivalent to the daily intake of 270, 870, and 960 mg/kg of rats [27]. In contemporary times, the incidence of arthritis was prevalent. Celecoxib, an effective therapeutic medicine, was more and more used as a new channel for the treatment of cancer and depression [28]. Opportunities for combining genistein and celecoxib have also increased. Based on the in vitro experiment, genistein inhibited the metabolic progress of celecoxib on RLM and HLM. From previous research, the IC50 on CYP2C9∗1 was ∼30 µM [16]. It was different with ∼11 µM. This difference was due to inconsistent enzyme and substrate selection. As we know, celecoxib had three metabolites, and CYP2C9 and CYP3A4 were responsible for the first step. Then, hydroxycelecoxib was metabolized by other enzymes. The research supposed that genistein inhibited the suppression of celecoxib. Moreover, CYP2C9∗3 (1075*A* > *C*) decreased the enzymatic activity to warfarin [29]. Sandberg et al. and Tang et al. founded that the *V*_max_/*K*_*m*_ ratio for hydroxycelecoxib was decreased by 34% (CYP2C9∗2) and 90% (CYP2C9∗3) in vitro. The AUC of celecoxib in CYP2C9∗1/∗3 and CYP2C9∗3/∗3 subjects was increased [30, 31]. Thus, many researches showed that gene polymorphism influenced the conversion of celecoxib. In the case of lower enzymatic activity, weak metabolism may exhibit toxicity at normal doses in combination of genistein and celecoxib. In our experiment of IC50 on 100 µM genistein-involved, CYP2C9∗3 was the most powerful suppression by the inhibitor (15%). According to CYP2C9 allele nomenclature, CYP2C9∗3 was changed in amino acid sequence that resulted Ile359 to Leu in substrate recognition site 5 [32]. CYP2C9∗3-induced allosteric protein showed defective ability in many clinical drugs including celecoxib [33, 34]. The individual who carried on that gene mutation would pay more attention on celecoxib administration with genistein. The data in Table 1 imply that the inhibitory effect of genistein was obvious to increasing dosage in vivo. When paclitaxel combinated with genistein, CL_z_/F decreased following the increase in AUC and C_max_. The pharmacokinetics changes of paclitaxel were consistent with our experiment [35]. CL_z_/F of celecoxib with 100 mg/kg genistein was significantly lower than 50 mg/kg with dose-dependent increasing *C*_max_. Celecoxib was accumulated too much in body because of genistein-influenced clearance of celecoxib. And suppression of genistein was dose dependent.

When normal metabolic process is suppressed, progress of pharmacokinetics will be influenced and homeostatic balance will be thrown off. In conclusion, genistein was an inhibitor of celecoxib both in vivo and in vitro. Genistein could inhibit the activity of liver microsomes, and celecoxib plasma levels were increased by decrement on metabolism of celecoxib with genistein. It is important to adjust the dosage of celecoxib to mitigate the adverse reaction which was induced by drug interaction. In the case of lower enzymatic activity, poor metabolic process may exhibit toxicity of celecoxib at normal doses when genistein is used in combination with celecoxib.

## Figures and Tables

**Table 1:** Main pharmacokinetics of celecoxib in three groups (*n* = 5).

Parameters	A	B	C
*C*_max_ (μg/l)	1380.55 ± 765.91	3240.00 ± 610.52^∗∗∗^	3756.71 ± 264.99^∗∗∗^
*t*_1/2_ (h)	4.34 ± 2.62	3.77 ± 0.76	2.93 ± 0.75
*T*_max_ (h)	2.60 ± 0.89	3.40 ± 0.89	3.40 ± 0.89
CL_z_/F (l/kg·h)	3.49 ± 1.37	2.03 ± 0.52^∗^	1.64 ± 0.20^∗∗^
AUC_(0–*t*)_ (μg/l·h)	10,821.66 ± 5555.93	25,675.06 ± 7187.40^∗∗^	30,576.35 ± 3593.70^∗∗∗^
AUC_(0–*∞*)_ (μg/l·h)	11,455.84 ± 6449.11	26,103.55 ± 7186.70^∗∗^	30,835.89 ± 3714.18^∗∗∗^
MRT_(0–*t*)_ (h)	6.70 ± 0.52	6.92 ± 0.60	6.46 ± 0.32
MRT_(0–∞)_ (h)	7.77 ± 1.70	7.32 ± 0.68	6.64 ± 0.42

Compared to group A (ANOVA), ^∗^*P* < 0.05; ^∗∗^*P* < 0.01; ^∗∗∗^*P* < 0.001.
